# Fatal crashes involving vehicles with driver warning systems: Identifying risk factors using a correlated random parameters ordered logit modeling approach

**DOI:** 10.1016/j.heliyon.2024.e33226

**Published:** 2024-06-19

**Authors:** Hardik Gajera, Srinivas S. Pulugurtha

**Affiliations:** Civil & Environmental Engineering Department, The University of North Carolina at Charlotte, 9201 University City Boulevard, Charlotte, NC 28223-0001, USA

**Keywords:** Driver warning systems, Crash, Fatal, Heterogeneity, Correlated random parameters

## Abstract

Recent advancements in vehicular technology are expected to enhance traffic safety by either warning the drivers or by automating the tasks related to driving to reduce the human driver's involvement. The driver warning systems (DWSs) are designed to warn drivers in unsafe situations such as forward collision, lane departure, or when changing lanes with vehicles in blind spot areas. Although these features are designed to enhance safety, recent crash data shows vehicles with these features are still getting involved in crashes, making it necessary to identify the contributing factors. Further, it also requires research to quantify the benefits of vehicles with one or multiple DWS in terms of safety during multivehicle crashes. This study presents a methodological framework to compare factors affecting fatal crashes involving vehicles with no, one and two DWSs. A three-step method is proposed to incorporate unobserved heterogeneity while modeling. Fixed parameter and correlated random parameter order logit models are employed. The results shows that correlated random parameters ordered logit model outperforms traditional fixed parameter ordered logit model. Vehicles equipped with DWSs are safer than vehicles without DWSs during wet or snowy road conditions, when the vehicle skids laterally or longitudinally, and at intersections. Vehicles with one or both DWSs can reduce drink-and-drive and speeding-related crash involvement than vehicles without DWSs. Female and elderly drivers are at a higher risk while driving a vehicle with one or both DWSs compared to driving a vehicle without DWSs, demanding vehicular modifications.

## 1 Introduction

The automotive industry, worldwide, has experienced a rapid increase in the adoption of driver warning systems (DWSs) over the past decade. The DWSs are designed to warn the driver in case of imminent collision. The DWSs, including blind spot monitoring (BSM), lane departure warning (LDW), and forward collision warning systems (FCWS), have gained popularity among manufacturers for their potential to enhance safety and mitigate crashes caused by human errors. BSM is a camera-based warning system that detects the presence of any vehicle in the blind spot and provides a warning to the driver. LDW is also a camera-based system that monitors the position of the vehicle within the driving lane and alerts the driver that the vehicle is departing the lane or approaching near the lane markers. FCWS is an NHTSA-recommended system that works based on radar detection. FCWS detects a potential collision with a vehicle in front and warns the driver. All the warning systems provide warnings using visual or audible devices depending on the make and model of the vehicle. Visual warnings are provided using lights indicating a particular warning feature is triggered, and audio systems generate a beeping sound to warn the driver to avoid dangerous maneuvers.

Despite advancements in vehicle technology, motor vehicle crashes remain a significant concern, with human errors contributing to around 94 % of crashes [1]. In 2019 alone, over 2.35 million people were injured or disabled, and approximately 36,000 lost their lives in fatal road crashes [2]. The economic impact of these crashes, including medical care and productivity losses, exceeded $75 billion in the United States [3]. In response to this safety challenge, DWSs are integrated into vehicles to improve traffic safety and reduce the number of crashes on the roads.

The potential benefits of DWSs in terms of improved safety and operational performance have raised expectations. As human error is the leading cause of motor vehicle crashes, additional warning provided by DWSs is expected to reduce crashes. The warning provided by DWSs could be either visual or acoustical, warning driver of potential hazard in advance and providing additional reaction time to avoid conflict. While adopting DWSs holds promise for improved safety, it is crucial to understand their real-world impact on traffic safety. Field tests during development can provide insights about variation in reaction time of drivers, but actual crash outcomes may differ from the anticipated benefits considering the variation in driver characteristics, vehicle make and models, and on and off-road characteristics. Therefore, a comprehensive analysis of real-world crash data is essential to understand the actual effectiveness of DWSs on traffic safety, particularly their impact on multivehicle fatal crashes. Research is needed to investigate how DWSs, such as FCWS and BSM, affect multivehicle fatal crashes, as their effects may differ from the anticipated benefits observed during field tests conducted during the development of these features.

The primary focus of this research is to investigate the effect of DWSs on multivehicle fatal crashes. The objective is to identify risk factors associated with crashes involving vehicles with no, one, and two DWSs. By analyzing real-world crash data, this research aims to provide valuable insights that can enhance traffic safety and guide future advancements in vehicular technologies.

A comprehensive safety assessment will help assess trends in multivehicle fatal crashes involving vehicles with one or two DWSs. The findings will help manufacturers and practitioners identify underlying risk factors and compare them with crashes involving vehicles without DWSs. This information can lead to appropriate modifications of existing DWSs and the development of new policies to improve road safety. Ultimately, the research findings will contribute to ongoing efforts to enhance road safety and create a safer transportation system for all road users. Leveraging the potential of DWSs, the automotive industry can significantly reduce traffic crashes and fatalities, making roads safer for everyone.

## 2 Literature review

Vehicular technologies and driver interactions are expected to evolve more in the next few decades than they have evolved in the past due to ongoing technological enhancements and research in the automobile industry [4,5]. Many researchers in the past have focused on identifying factors influencing single and multivehicle fatal crashes [[6], [7], [8], [9]]. The identified risk-causing factors related to on-road and off-road characteristics in the existing literature are the dimension of the median [6], day of the week [7], side traffic barriers [8], speed limit [9], road infrastructure [9], highway class [10], road alignment [7], socio-demographic characteristics near the crash location [11], traffic control [7], and adverse weather conditions [12]. Besides, some researchers also evaluated the effect of red-light cameras [13], road surface [14], intersection type [15], and annual average daily traffic (AADT) [15,16] on crashes at intersections. These studies focused on identifying the factors related to crashes or conducting a before and after analysis to determine the effect on traffic safety. In addition to the on-road and off-road characteristics, vehicular characteristics are also amongst factors affecting crash occurrence as per the existing literature [1,4].

Vehicular characteristics (presence of advanced features, safety standards and ratings, size, and type of vehicle) are also among the factors influencing crash occurrence and injury severity. In addition, driver characteristics also affect fatal crash occurrences. Human errors are among the most frequent causes of crashes in the United States [1]. The causes of human error primarily vary depending on driver characteristics such as age, gender, and driver experience. Teen drivers are generally found to be aggressive and inexperienced, making them more vulnerable to crashes [4]. Findings from the research on teen drivers indicate that the crash rate per mile driven and crash rate per number of license holders for teen drivers are higher than for adults [17]. In contrast, elderly drivers are found to have poor reaction time, ability to divide attention between multiple tasks, and vision [18], due to which their probability of getting involved in a crash is higher compared to young drivers [19]. In addition to age, several other factors, such as gender, distracted driving, and driving under the influence of alcohol or drugs, also influence the likelihood of getting involved in a crash. Therefore, considering driving behavior while analyzing crash data is necessary to get useful insights about variations in crash risk.

To analyze the effects of vehicles on safety, several researchers used different parametric as well as non-parametric models such as the negative binomial model [20], spatial autoregressive model [20], modified negative binomial regression [21], multivariate adaptive regression [20], bootstrap-based binary logistic regression [22], fixed-parameters logit model [23], random parameters logit model [[23], [24], [25]], and correlated random parameters model [26]. These modeling techniques were used to analyze crash data and identify factors affecting crash severity. Amongst these modeling techniques, the correlated random parameters ordered model is identified to be superior compared to other counterparts [26]. Furthermore, use of modeling techniques considering random parameters to determine the effectiveness of DWSs would also provide detailed insights on variability of factors affecting crashes with the characteristics that are individual crash specific such as driver related factors.

Existing literature contains a plethora of research on DWSs. They provide a warning to the drivers departing their travel lane or making dangerous maneuvers, such as changing lanes while having a vehicle in a blind spot area or late-braking while having vehicles in front. The FCWS warns the driver of potential forward collision conditions, reducing the likelihood of getting involved in rear-end collisions [27]. The BSM system, also known as the side view assist system, warns the driver when any vehicle or object is in the vehicle's blind spot area [27]. The LDW system warns drivers of conditions when a vehicle departs a lane, generally affecting single-vehicle roadside departure or lane departure-related crashes.

Reagan et al. (2018) conducted a study to identify the utilization of different warning features [28]. Their results showed that LDW is turned on all the time by 20 % of drivers. However, BSM and FCWS or front crash prevention systems were turned on all the time by 99 % and 93 % of the drivers. Considering the lower utilization of the LDW feature, only BSM and FCWS are considered in this research. In past, several researchers examined the effects of individual DWSs on safety. Examples include studies to identify the effect of the FCWS [27] and crash avoidance technology [29] on safety. Fitch et al. (2014) investigated the effectiveness of using various DWSs in multiple near-crash scenarios with FCW and LDW systems [30]. The results showed that vehicles with multiple DWSs features are safer than others. Due to warnings provided by vehicles with DWSs during potential crash conditions, they are driving the existing market because of the increasing emphasis on safety and operations. However, before transiting to the scenario with completely automated vehicles, it is necessary to identify the safety effect of individual and combined DWSs and their combinations to improvise the technology and ultimately enhance the safety effectiveness of these vehicles.

Most previously conducted studies related to individual DWS were carried out using simulation analysis. The simulation analysis includes either microsimulation software or driving simulators [31] to mimic the behavior of AVs and identify their effect on traffic operations and safety. The review of these studies showed mixed results, primarily due to varying assumptions and considerations.

The effectiveness of DWSs also varies depending on factors related to road geometry, crash occurrence, and vehicle. A comprehensive safety assessment using crash data and considering all the factors affecting fatal crashes helps gain insights into the factors affecting fatal crashes involving vehicles equipped with one or more DWSs.

This research focuses on bridging the research gap by identifying risk factors influencing fatal crashes involving vehicles with and without DWS and synthesizing the difference in factors affecting fatal crashes involving vehicles with and without DWS. The methodological framework used in this study incorporates three significant aspects of unobserved heterogeneity while modeling, making the model estimates more interpretable and accurate.

## 3 Methodology

The study area should be selected to get the maximum number of samples, and it should be large enough to consider the spatial effects and the effect of varying geometry depending on the locations and road types. In this study, the United States is considered as the study area.

In order to identify the effect of various DWSs on fatal crashes, crash data was collected from the Fatality Analysis Reporting System (FARS) database. Further, the VINs of all the vehicles were used, and data on DWSs were retrieved from the National Highway Traffic Safety Administration (NHTSA) database. The VINs data showed that the number of vehicles with DWSs, such as BSM and FCWS, was much lower in 2016. Therefore, data from the year 2016–2020 was considered for the analysis. The FARS database contains information related to factors affecting crashes, vehicles involved in the crashes, and pedestrian involvement in the crashes. To obtain information about DWSs, the data processing framework proposed by Gajera et al. (2023) was used to retrieve DWSs-related information from the NHTSA database using VINs available in FARS [5].

### 3.1 Data processing

The raw FARS data are available in different files related to crashes, vehicles, pedestrians, and other characteristics. Initially, the obtained data in separate files for each year from 2016 to 2020 was linked using the assigned ID and year, the standard fields in all files of the FARS database. The data from each year were combined into a complete dataset. Some samples had missing values or not-reported data, which were removed using filtering technique considering the fact that less than 2 % of the samples had missing values. Moreover, the bias due to eliminated samples is not expected to affect the model results as the overall objective of the study is to compare vehicles with different DWSs and hence an implicit assumption is that both (vehicles with and without DWSs) have missing values which are filtered. However, the methodology adopted in this research recommends consideration of potential bias in case if larger proportion of samples have missing or null values.

The VINs of all vehicles in the combined dataset were extracted as a separate file. A Python script was used to extract data related to DWSs. Initially, a loop was created for extraction to identify all the vehicles' information in a single trial. The loop looks up a single VIN in the input list and connects it with the information from the VIN dataset of NHTSA. The loop then returns the information of all DWSs as a list. Further, the information in the list is transformed into a single raw data frame in the same loop. Finally, the data, including the VINs and the information about DWSs in the vehicle, was combined with the FARS data.

**Fig. 1:**
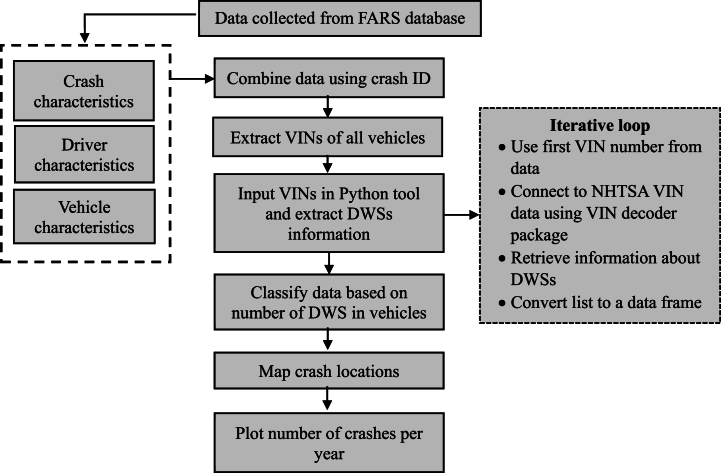
Data processing framework.

The obtained dataset contained information about DWSs, such as LDW, FCWS, and BSM. The dataset showed that various regions had negligible crashes involving vehicles with DWSs compared to crashes involving vehicles without a DWS. The samples involving one or more vehicles equipped with different DWSs were also identified and considered as separate data points in the analysis. Before modeling, the samples with unknown or unidentified values were eliminated using a filtering technique to reduce the redundancies in the result. The data processing framework used in this study is summarized as shown in Fig. 1.

Using VINs and the NHTSA VIN decoder, generic information on the availability of DWSs of vehicles in fatal crashes was extracted. However, this has limitations that need to be handled meticulously. For instance, for most DWSs, the VIN decoder provides DWS availability for the vehicle as “Standard,” “Optional,” or “Not Available,” or has missing values. The data varies based on the vehicle's make, model, year, and trim level. If the code is “Standard,” it is known that the DWS feature is on the vehicle. If the code is “Not Available,” DWS is unavailable on the vehicle. Only data with the VIN code “Standard” were used in this study. Further, only multivehicle crashes were considered in the analysis as FCWS and BSM features are specifically designed to improve safety in forward impact and lane change-related crashes, which are often multivehicle, and considering single vehicle and pedestrian crashes may result in biased estimates.

**Fig. 2:**
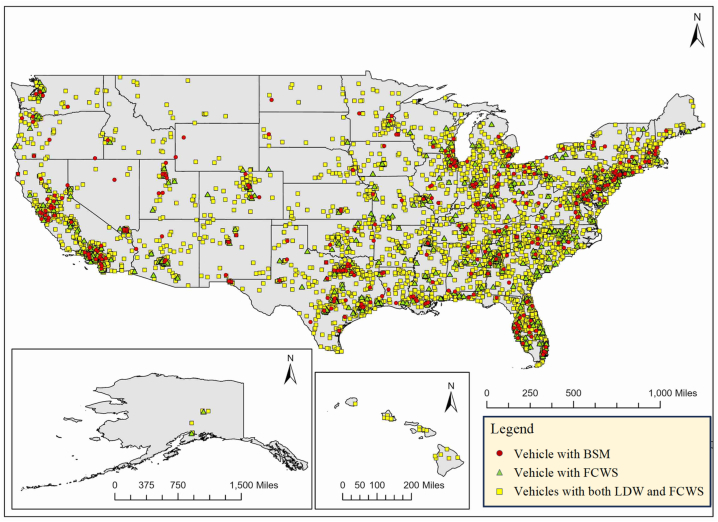
Crashes involving vehicles with FCWS and BSM.

To visualize the spatial trends in crashes involving vehicles with one or multiple DWS, the location of crashes is plotted as shown in Fig. 2.

**Fig. 3:**
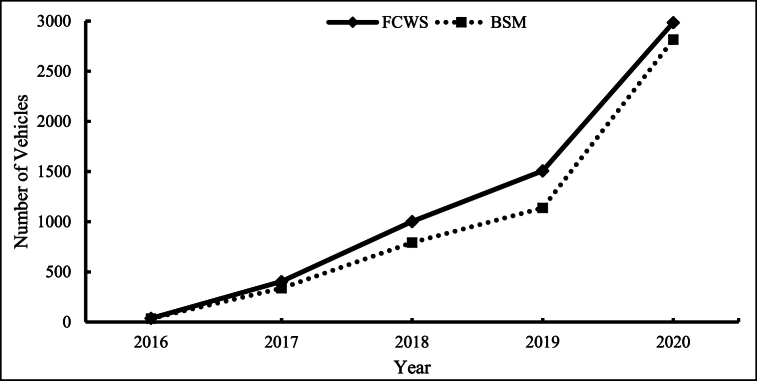
Variation in number of crashes involving vehicles with FCWS and BSM.

The major clusters, including vehicles with one or multiple DWSs involved in fatal crashes, are in the eastern regions, as shown in Fig. 2. The number of crashes involving vehicles with DWSs in central and mountain regions is low compared to the east and west coasts. As the crashes vary spatially, it is necessary to account for spatial heterogeneity due to the varying locations of crashes. Further, the number of crashes per year is estimated to visualize the temporal trends. The number of crashes involving vehicles with DWS from 2016 to 2020 is shown in Fig. 3.

The fatal crash involvement of vehicles with DWSs shows an increasing trend over the study years. The number of vehicles with FCWS and BSM gradually increased from negligible number of crashes in 2016 to 1500 and 1000 crashes in 2019. From 2019 to 2020, a sudden increase in the number of crashes is observed, potentially due to the increasing penetration of vehicles with these features. The trend shown in Fig. 3 shows temporal heterogeneity in crash data, and it is necessary to account for it while modeling.

In addition to spatial and temporal heterogeneity, the dataset has heterogeneity due to varying driver behaviors. Because each driver is different in experience, age, gender, and driving habits, it is necessary to account for heterogeneity due to driving behavior while modeling. To account for unobserved heterogeneity while modeling, a methodological framework is proposed in this research, which breaks down heterogeneity into three aspects and accounts for it at various stages of data processing and modeling.

From the initial descriptive statistics, it is noted that the number of vehicles in the dataset with FCWS, BSM, or both was less than 3 %. Therefore, to compare vehicles with and without DWSs, it is necessary to select samples from crashes involving vehicles without DWSs. The nearest neighbor analysis is recommended in the existing literature for sampling in cases where the sample size is too large [32]. In the nearest neighbor analysis, the number of nearest neighbors from locations of crashes involving vehicles with DWSs is optimized to identify the optimum number of crashes for the sample. The use of nearest-neighbor sampling also ensures that the comparison groups (crashes involving vehicles with and without DWSs) are in spatial proximity, as past research findings indicate that crashes are correlated spatially [33]. Therefore, the nearest neighbor analysis was used for sampling crashes involving vehicles without DWSs. The optimum number of nearest neighbors is identified as three, and the maximum distance of the third neighbor in the dataset is estimated to be 3167 feet.

Fatal crash data from 2016 to 2020 was used in this study. Therefore, to account for temporal heterogeneity due to variation in time, the temporal variable “Year of a crash” in the form of linear effects of time elapsed was incorporated in modeling. The variable was coded as 1 for 2016, 2 for 2017, 3 for 2018, 4 for 2019, and 5 for 2020. Doing so provides estimates for the year, which describes the variation in the probability of crash occurrence over the study years.

A correlated random parameters modeling technique is employed to account for heterogeneity due to varying driving behaviors. Variables related to driving behavior parameters such as age, gender, and drinking and driving were kept as random parameters in all the models, allowing them to vary across observations. The correlated random parameters model also accounts for correlation amongst considered random parameters, making it a more appropriate method in this study.

### 3.2 Fixed and correlated random parameters ordered logit models

Ordered probability models, such as ordered probit or logit models, are regression models used when the dependent variable has three or more categories with a meaningful order [34]. In this context, the variable of interest is the number of DWSs in a vehicle involved in a fatal crash. These DWSs provide varying levels of safety effectiveness, making it essential to consider the ordered nature of this variable.

The order of the categories is determined based on the information and warnings provided to drivers during different driving tasks, which correspond to the number of features available in the vehicle. To analyze this relationship, a fixed parameter ordered logit model is developed, offering the flexibility to calculate marginal effects (direct and cross marginal effects) for both continuous and indicator variables. Additionally, this fixed parameters model is computationally less demanding compared to random parameters models, making it a suitable starting point for the analysis.

Assuming that there are *N* observations indexed by *i* (*i = 1, 2, 3, …, N*) and the number of DWSs in a vehicle *i* is indexed by *k* (*k = 0, 1, 2, …, S,* where *S =* 2), the variable *k* takes the value 0 for ‘vehicles without FCWS or BSM,’ 1 for ‘vehicles with either FCWS or BSM,’ and 2 for ‘vehicles with both FCWS and BSM.'

In the ordered outcome framework, the actual number of features in vehicle ($Y_{i}$) is assumed to be associated with a continuous latent variable (${Y_{i}}^{*}$). The latent propensity is represented as a linear function in Equation (1).

(1) $${Y_{i}}^{*}=\beta X_{i}+\varepsilon_{i}$$

where $X_{i}$ is the vector of observed independent variables, β is the vector of estimable parameters and ε is the random error term. The latent propensity is mapped to the actual categories of dependent variable by the $\psi_{k}$ thresholds ($\psi_{k}=-\infty and\;\psi_{k}=\infty$). In equation (1), the term β is assumed to be fixed across observations. Therefore, Equation (1) converges to a fixed parameters ordered logit model.

As the driver characteristics such as driver fatigue, drivers' prior experience and expertise in driving, driver's age and gender, and their reaction times account for possible unobserved heterogeneity, random parameters model is a vital option for modeling as it facilitates incorporating unobserved heterogeneity in dataset while modeling [24,[35], [36], [37]]. The random parameters ordered logit model assumes parameters in the model to vary across the observation, and the variation follows a specific distribution. In addition, it also provides the flexibility to vary the mean and variance across observations of the dataset [37].

To relax the fixed parameters assumptions, i.e., the effect of (β) is the same across observations, a correlated random parameters approach was employed, wherein the (β) was allowed to vary systematically across each observation. The correlated random parameters can be expressed as shown in Equation (2) [38].

(2) $$\beta_{i}=\beta +\Omega \varphi$$

where β is the mean of the random parameter, Ω is a lower triangular Cholesky matrix containing information about covariances and it also accounts for possible correlations amongst the coefficients, and φ is a column vector of independent standard normally distributed variables. It is assumed that $\beta_{i}$ follows a multivariate normal distribution with mean β and a covariance matrix $\Omega \Omega^{\prime }$. In this study, unrestricted form of Cholesky matrix is used, which allows to capture correlations between multiple random parameters.

One of the practical limitations of this modeling framework is that the effect of the independent variables on latent propensity cannot quantify the effect of each variable on the probability of various categories of dependent variable [39]. Therefore, average marginal effects are computed to obtain the effect of explanatory variables for each ordinal outcome. For an explanatory variable $X_{i}$, the average marginal effects are estimated using the difference in the estimated probabilities when the variable is changing from zero to one, while all other variables ($\bar{X_{l}})$ are equal to the average values of the sample observations. The average marginal effects for the indicator variable is mathematically expressed as shown in Equation (3) [39].

(3) $$AME=P\left(Y=k|\bar{X_{l}},X_{i}=1\right)-P\left(Y=k|\bar{X_{l}},X_{i}=0\right)$$

The random function can follow any distribution, such as normal, lognormal, uniform, Weibull, or triangular distribution. The selection of distribution influences the accuracy of the results. The existing literature shows that using the normal distribution for random functions provides better accuracy of the results. Therefore, random parameters are assumed to be normally distributed. The random values considered for modeling may affect the outcomes, due to which multiple draws of the random sample must be tested for specific random functions.

The existing literature suggests that the Halton sequence approach is one of the best ways to draw random values [40,41]. The Halton sequence is a sequence of dimensional numbers, and it is generated using the deterministic method. The Halton numbers are designed to give fairly even coverage through the domain of the selected distribution [42]. Thus, Halton draws are used in this study instead of random draws. A recent study by Hamed et al. (2022) showed that using 900 Halton draw provides stable model estimates and therefore is considered in this study [43].

## 4 Results

### 4.1 Descriptive statistics

A descriptive analysis was conducted to identify the frequency distribution across different categories of dependent and independent variables. The frequency and percentage of samples across each category of all variables are shown in Table 1.

**Table 1:** Descriptive statistics.

Variable		Frequency (Percentage)
**DWSs**		
No FCWS or BSM		6366 (72.696)
Either FCWS or BSM		1628 (18.591)
Both FCWS and BSM		763 (8.713)
**Driver characteristics**		
Age	Less than 24 years	1392 (15.896)
	≥24, ≤ 40 years	2765 (31.575)
	>40, ≤ 65 years	3112 (35.537)
	Greater than 65 years	1488 (16.992)
Drink and drive related		923 (10.54)
Not related to drink and drive		7834 (89.46)
Gender	Female	2781 (31.757)
	Male	5976 (68.243)
**Road characteristics**		
Area type	Urban	5248 (59.929)
	Rural	3509 (40.071)
Functional class	Interstate	1149 (13.121)
	Freeway or expressway	408 (4.659)
	Principal arterial	3394 (38.758)
	Minor arterial	2062 (23.547)
	Major collector	1029 (11.751)
	Minor collector	196 (2.238)
	Local	519 (5.927)
Intersection		5231 (59.735)
Non-intersection		3526 (40.265)
Number of lanes	No traffic way access	84 (0.959)
	One lane	85 (0.971)
	Two lanes	5001 (57.109)
	Three lanes	1281 (14.628)
	Four lanes	1112 (12.698)
	Five lanes	842 (9.615)
	Six lanes	209 (2.387)
	Seven or more lanes	143 (1.633)
Work zone		254 (2.901)
No work zone		8503 (97.099)
**Crash characteristics**		
Light condition	Daylight	5387 (61.517)
	Dark	3008 (34.35)
	Dawn	173 (1.976)
	Dusk	189 (2.158)
Pre-crash stability	Tracking	6682 (76.305)
	Skidding laterally	148 (1.69)
	Skidding longitudinally	103 (1.176)
	Not specific	1824 (20.829)
Surface condition	Dry	7483 (85.452)
	Wet	1036 (11.831)
	Ice, snow, mud, dirt, oil, or water	238 (2.718)
Season	Winter	1863 (21.274)
	Spring	1939 (22.142)
	Summer	2511 (28.674)
	Fall	2444 (27.909)
Speeding		978 (11.168)
Not speeding		7779 (88.832)
Time of the day	12 a.m.–3 a.m.	597 (6.817)
	3 a.m.–6 a.m.	524 (5.984)
	6 a.m.–9 a.m.	947 (10.814)
	9 a.m. to 12 p.m.	1106 (12.63)
	12 p.m.–3 p.m.	1500 (17.129)
	3 p.m.–6 p.m.	1815 (20.726)
	6 p.m.–9 p.m.	1297 (14.811)
	9 p.m. to 12 a.m.	971 (11.088)
Weather condition	Clear	6576 (75.094)
	Cloudy	1281 (14.628)
	Rain	668 (7.628)
	Snow, fog/smoke/smog, or other adverse condition	232 (2.649)
Manner of collision	Head-on	2439 (27.852)
	Rear-end	1799 (20.544)
	Angle	3825 (43.679)
	Sideswipe - opposite direction	389 (4.442)
	Sideswipe - same direction	305 (3.483)

As per the descriptive statistics, most of the drivers involved in fatal crashes fall within the age range of 24–65 years, with the highest proportion being in the 40–65 years category. This indicates that middle-aged drivers are more frequently involved in such crashes. A significant proportion of these crashes (10.54 %) involve drinking and driving. Male drivers account for a higher percentage (68.243 %) than their female counterparts (31.757 %), indicating potential disparities in driving behavior and a propensity for risk-taking.

Further, crashes are found to be more prevalent in urban areas (59.929 %) compared to rural areas (40.071 %). In case of road type, crashes primarily occurred on principal and minor arterials. Intersections contribute significantly to crashes, accounting for 59.735 % of the incidents. Roads with two lanes are the most common (57.109 %), and the number of crashes on roads with different number showed significant variation, demanding further analysis.

Crash characteristics included in this study highlights the factors contributing to the crashes. Most crash incidents happen under daylight conditions (61.517 %) and on dry road surfaces (85.452 %). The most frequent manner of collision is classified under the “Angle” category, representing 43.679 % of crashes. Driver behavior is also a significant factor, with speeding being involved in 11.168 % of crashes. Additionally, crashes are more prevalent during peak afternoon hours, specifically between 3 p.m. and 6 p.m., highlighting the importance of considering the time of day in crash prevention strategies.

### 4.2 Model fit outcomes

Fixed and correlated random parameter ordered logit models were developed. The goodness-of-fit for both models were assessed using Log-Likelihood and McFadden pseudo-R-squares [44] and compared to determine the better fitting model. The results indicate that the Log-likelihood value for the correlated random parameters model is −6124.668, and the log-likelihood value of the fixed parameters model is −6138.570, indicating that the correlated random parameters model is better fitted. Moreover, the McFadden Pseudo R-squared value for the correlated random parameters model is 0.076, which is higher than the 0.074 McFadden Pseudo R-squared value for the fixed parameters model, supporting the superiority of the former models in explaining the data. Considering the better performance of the correlated random parameters model, the results for the same are documented. The model estimates for the correlated random parameters model are shown in Table 2.

**Table 2:** Correlated random parameters ordered logit model estimates.

Variables	Coefficient	Standard error	*z-*value	*p-*value
Constant	−3.898	0.175	−22.320	<0.001
Year	0.584	0.026	22.310	<0.001
Area type (Urban)	0.240	0.061	3.910	<0.001
Season (Winter)	−0.086	0.081	−1.060	0.288
Season (Spring)	−0.050	0.075	−0.660	0.509
Season (Fall)	0.171	0.069	2.470	0.014
Time of the day (12 a.m.–3 a.m.)	−0.140	0.164	−0.860	0.392
Time of the day (3 a.m.–6 a.m.)	−0.204	0.164	−1.250	0.212
Time of the day (9 a.m.–12 p.m.)	0.306	0.115	2.650	0.008
Time of the day (12 p.m.–3 p.m.)	0.227	0.110	2.070	0.039
Time of the day (3 p.m.–6 p.m.)	0.175	0.105	1.660	0.096
Time of the day (6 p.m.–9 p.m.)	0.106	0.122	0.870	0.385
Time of the day (9 p.m.–12 a.m.)	−0.047	0.147	−0.320	0.748
Manner of collision (Head-on)	0.215	0.077	2.790	0.005
Manner of collision (Rear-end)	0.029	0.082	0.360	0.721
Manner of collision (Sideswipe - opposite direction)	0.114	0.132	0.860	0.388
Manner of collision (Sideswipe - same direction)	0.247	0.153	1.610	0.107
Speeding	−0.139	0.093	−1.490	0.136
Number of lanes (No trafficway access)	0.318	0.274	1.160	0.245
Number of lanes (one lane)	0.304	0.257	1.180	0.237
Number of lanes (Three lanes)	0.025	0.083	0.300	0.766
Number of lanes (Four lanes)	0.242	0.085	2.840	0.005
Number of lanes (Five lanes)	0.211	0.094	2.230	0.026
Number of lanes (Six lanes)	0.165	0.174	0.950	0.341
Number of lanes (Seven or more lanes)	0.268	0.214	1.260	0.209
Surface condition (Wet)	−0.048	0.136	−0.350	0.725
Surface condition (Ice, snow, mud, dirt, oil or water)	−0.652	0.266	−2.450	0.014
Pre-crash stability (Skidding laterally)	−0.157	0.215	−0.730	0.466
Pre-crash stability (Skidding longitudinally)	−0.483	0.282	−1.710	0.087
Pre-crash stability (Not specific)	−0.263	0.073	−3.600	<0.001
Functional class (Interstate)	0.284	0.093	3.060	0.002
Functional class (Freeway or expressway)	0.376	0.127	2.970	0.003
Functional class (Minor arterial)	−0.096	0.069	−1.380	0.167
Functional class (Major collector)	−0.123	0.092	−1.330	0.184
Functional class (Minor collector)	0.173	0.177	0.980	0.328
Functional class (Local)	0.058	0.120	0.480	0.631
Intersection	−0.138	0.071	−1.940	0.053
Work zone	0.160	0.158	1.020	0.310
Light condition (Dark)	0.248	0.109	2.270	0.023
Light condition (Dawn)	−0.175	0.215	−0.810	0.415
Light condition (Dusk)	0.182	0.195	0.930	0.350
Weather condition (Cloudy)	−0.041	0.079	−0.520	0.603
Weather condition (Rain)	0.011	0.165	0.060	0.949
Weather condition (Snow, fog/smoke/smog, or other adverse condition)	0.494	0.194	2.540	0.011
**Means of random parameters**				
Drink and drive	−0.429	0.101	−4.250	<0.001
Gender (Female)	0.684	0.055	12.370	<0.001
Age (Less than 24 years)	−0.296	0.084	−3.520	<0.001
Age (>40, ≤ 65 years)	−0.347	0.068	−5.100	<0.001
Age (Greater than 65 years)	0.196	0.078	2.500	0.013
**Diagonal elements of Cholesky matrix**				
Drink and drive	0.453	0.092	4.900	<0.001
Gender (Female)	0.779	0.051	15.180	<0.001
Age (Less than 24 years)	0.387	0.069	5.650	<0.001
Age (>40, ≤ 65 years)	0.383	0.047	8.210	<0.001
Age (Greater than 65 years)	0.197	0.060	3.310	0.001
**Below diagonal elements of Cholesky matrix**				
Gender (Female) - Drink and drive	0.242	0.050	4.830	<0.001
Age (Less than 24 years) - Drink and drive	−0.619	0.073	−8.510	<0.001
Age (Less than 24 years) - Gender (Female)	−0.484	0.071	−6.830	<0.001
Age (>40, ≤ 65 years) - Drink and drive	−0.890	0.054	−16.560	<0.001
Age (>40, ≤ 65 years) - Gender (Female)	0.194	0.050	3.910	<0.001
Age (>40, ≤ 65 years) - Age (Less than 24 years)	−0.385	0.047	−8.250	<0.001
Age (Greater than 65 years) - Drink and drive	−0.209	0.062	−3.340	0.001
Age (Greater than 65 years) - Gender (Female)	−0.912	0.066	−13.820	<0.001
Age (Greater than 65 years) - Age (Less than 24 years)	0.030	0.059	0.510	0.612
Age (Greater than 65 years) - Age (>40, ≤ 65 years)	−0.253	0.060	−4.240	<0.001
**Threshold parameters for probabilities**				
Threshold parameters for probabilities	1.644	0.039	42.530	<0.001

The model holds the first assumption of Ordered Logit models that the dependent variable is ordinal as the vehicles with zero, one and two DWSs is considered as the dependent variable, which follows natural order. One of the drawbacks of fixed parameters model is that the multicollinearity between different variables. The fixed parameter model does violate the assumptions however all variables are still considered in the model to facilitate the comparison with random parameters model which does not violate the multicollinearity assumption.

Most variables are statistically significant at a 90 % or higher confidence level. To ensure the identifiability of the classification thresholds, the threshold delineating vehicles without any DWS and vehicles with one DWS is fixed to zero. The threshold delineating vehicles with one and two DWSs is identified to be 1.644. The coefficients show the loading for a particular variable in the model, which determines the number of DWSs in the vehicles using the established thresholds. A negative coefficient value indicates that the presence of a particular variable is shifting the predictions toward vehicles without any DWS. In contrast, a positive coefficient indicates the presence of a specific variable adding to the estimates toward one or more DWSs.

The p-value of all the means of random parameters is less than 0.013, indicating that all the random parameters are highly significant. The diagonal elements of the Cholesky matrix and the below diagonal elements of the Cholesky matrix indicate the correlation between various variables and the significance of the correlation. All other possible combinations of random parameters are significantly correlated except for ages greater than 65 years and less than 24 years.

Overall, incorporating a correlated parameters model, which considers the correlation between variables, yields better results as the modeling framework considers the case specific variables such as driver related attributes and also the correlation amongst the variables, relaxing the model from multicollinearity assumption of ordered logit models. The model coefficients are not directly interpretable regarding their contribution to the crash occurrence. Therefore, partial effects were obtained to facilitate the comparative analysis and determine the effect of each variable on different categories of dependent variable, as shown in Table 3. The authors recommend using partial effects as measure of comparison between the categories of dependent variables however consideration of the same for prediction is outside the scope of this research and is also considered as the topic for further research.

**Table 3:** Partial effects.

Variables	Y = 0		Y = 1		Y = 2	
	Partial effect	*p-*value	Partial effect	*p-*value	Partial effect	*p-*value
Year	−0.099	<0.001	0.071	<0.001	0.028	<0.001
Area type (Urban)	−0.040	<0.001	0.029	<0.001	0.011	<0.001
Season (Winter)	0.014	0.281	−0.010	0.282	−0.004	0.277
Season (Spring)	0.008	0.506	−0.006	0.507	−0.002	0.504
Season (Fall)	−0.030	0.015	0.021	0.015	0.008	0.017
Time of the day (12 a.m.–3 a.m.)	0.023	0.374	−0.017	0.378	−0.006	0.366
Time of the day (3 a.m.–6 a.m.)	0.033	0.188	−0.024	0.192	−0.009	0.176
Time of the day (9 a.m.–12 p.m.)	−0.055	0.012	0.039	0.011	0.016	0.017
Time of the day (12 p.m.–3 p.m.)	−0.040	0.047	0.028	0.044	0.012	0.053
Time of the day (3 p.m.–6 p.m.)	−0.030	0.106	0.022	0.103	0.009	0.112
Time of the day (6 p.m.–9 p.m.)	−0.018	0.395	0.013	0.392	0.005	0.401
Time of the day (9 p.m.–12 a.m.)	0.008	0.746	−0.006	0.746	−0.002	0.744
Manner of collision (Head-on)	−0.037	0.007	0.027	0.006	0.011	0.008
Manner of collision (Rear-end)	−0.005	0.722	0.004	0.722	0.001	0.723
Manner of collision (Sideswipe - opposite direction)	−0.020	0.401	0.014	0.398	0.006	0.409
Manner of collision (Sideswipe - same direction)	−0.044	0.129	0.031	0.122	0.013	0.145
Speeding	0.023	0.123	−0.016	0.126	−0.006	0.117
Number of lanes (No trafficway access)	−0.058	0.282	0.041	0.269	0.018	0.310
Number of lanes (one lane)	−0.056	0.272	0.039	0.260	0.017	0.299
Number of lanes (Three lanes)	−0.004	0.767	0.003	0.767	0.001	0.768
Number of lanes (Four lanes)	−0.043	0.007	0.030	0.006	0.013	0.009
Number of lanes (Five lanes)	−0.037	0.033	0.026	0.031	0.011	0.039
Number of lanes (Six lanes)	−0.029	0.362	0.021	0.356	0.009	0.375
Number of lanes (Seven or more lanes)	−0.049	0.239	0.034	0.229	0.014	0.261
Surface condition (Wet)	0.008	0.722	−0.006	0.723	−0.002	0.721
Surface condition (Ice, snow, mud, dirt, oil or water)	0.091	0.002	−0.067	0.003	−0.024	0.001
Pre-crash stability (Skidding laterally)	0.025	0.446	−0.018	0.450	−0.007	0.435
Pre-crash stability (Skidding longitudinally)	0.071	0.045	−0.052	0.050	−0.019	0.032
Pre-crash stability (Not specific)	0.043	<0.001	−0.031	<0.001	−0.012	<0.001
Functional class (Interstate)	−0.051	0.004	0.036	0.003	0.015	0.005
Functional class (Freeway or expressway)	−0.070	0.006	0.049	0.005	0.021	0.010
Functional class (Minor arterial)	0.016	0.161	−0.011	0.162	−0.004	0.158
Functional class (Major collector)	0.020	0.172	−0.015	0.175	−0.006	0.166
Functional class (Minor collector)	−0.031	0.349	0.022	0.343	0.009	0.363
Functional class (Local)	−0.010	0.635	0.007	0.634	0.003	0.638
Intersection	0.023	0.051	−0.017	0.052	−0.007	0.051
Work zone	−0.028	0.329	0.020	0.324	0.008	0.342
Light condition (Dark)	−0.043	0.026	0.031	0.025	0.012	0.029
Light condition (Dawn)	0.028	0.391	−0.020	0.396	−0.008	0.379
Light condition (Dusk)	−0.032	0.372	0.023	0.366	0.009	0.387
Weather condition (Cloudy)	0.007	0.600	−0.005	0.600	−0.002	0.598
Weather condition (Rain)	−0.002	0.949	0.001	0.949	0.001	0.949
Weather condition (Snow, fog/smoke/smog, or other adverse condition)	−0.094	0.022	0.065	0.017	0.029	0.037
Drink and drive	0.066	<0.001	−0.048	<0.001	−0.018	<0.001
Gender (Female)	−0.124	<0.001	0.087	<0.001	0.037	<0.001
Age (Less than 24 years)	0.047	<0.001	−0.034	<0.001	−0.013	<0.001
Age (>40, ≤ 65 years)	0.057	<0.001	−0.041	<0.001	−0.016	<0.001
Age (Greater than 65 years)	−0.034	0.016	0.024	0.015	0.010	0.019

The partial effects shown in Table 3, along with the p-value, indicate that the effect of variables on crashes involving vehicles with one or more DWSs varies compared to vehicles without DWSs. The negative values of partial effects indicate that vehicles without DWSs have less probability of getting involved in a fatal multi-vehicle crash according to particular variables. The variable year is added to the model to account for the temporal heterogeneity. It shows that the fatal crash occurrence probability for vehicles without DWS decreased over the study years. However, the probability of fatal crash occurrence increased for vehicles with one or more DWSs. Considering that the penetration of vehicles with DWSs increased over the study years in the data, it is apparent that the partial effect for the variable “year of a crash” is positive for one or more than one DWS.

The road geometry and location-related variables such as urban areas, number of lanes (one to seven or more lanes), and functional class (intestate, freeways, expressways, and local) show that vehicles with one or more DWSs have a higher probability of crash occurrence. In contrast, vehicles with one or more DWSs have a lower probability of crash occurrence than vehicles without DWSs on major collector, major arterial, and minor arterial roads, and at intersections. Vehicles with one or more DWSs have a lower probability of crash occurrence during night time, in winter and spring seasons, in conditions when a driver is speeding, in wet, ice or snow, or muddy road surface conditions, in conditions when a vehicle is skidding laterally or longitudinally, during dawn light conditions, and in cloudy weather conditions. Similarly, vehicles with one or more DWSs are safer in drink and drive-related crashes than those without DWSs.

The driver characteristics-related variables are highly significant and show that the probability of crash occurrence is higher for females when driving vehicles with one or more DWSs. Similarly, the probability of crash occurrence when driving a vehicle with DWSs is higher for elderly drivers (age greater than 65 years). In contrast, the probability of fatal crashes is lower when driving vehicles with DWSs for teen and young drivers. Potential reasons for the same could be the familiarity of younger drivers with warning systems and their reaction time after receiving alerts from the DWS. Although the model fit statistics clarifies that the correlated random parameters model edges the fixed parameter model with slight variation in pseudo r-square and log-likelihood statistics, the correlated random parameters model relax the multicollinearity assumption and hence provides naturally better fit by incorporating case specific heterogeneity and correlating within modeling framework.

## 5 Conclusions

A methodological framework to identify DWSs in a vehicle using VINs and incorporating unobserved heterogeneities while modeling is proposed in this study to identify the factors affecting fatal crashes involving vehicles with zero, one, and two DWS. The method includes collecting fatal crash data from 2016 to 2020 and retrieving information about DWSs in the involved vehicles. Spatial maps and temporal variation plots were developed to visualize crashes' spatial and temporal patterns. The trends observed showed variations in crashes across different regions in the United States, and the number of crashes involving vehicles with particular DWSs increased over the study years. Therefore, nearest neighbor analysis was conducted to eliminate spatial heterogeneity and ensure a fair comparison.

Descriptive analysis and modeling using fixed and correlated random parameters ordered logit models were performed to determine the effects of DWSs on crash occurrences. Further, a linearly varying temporal variable was incorporated into the model to account for temporal heterogeneity. All driver-related parameters were considered random for heterogeneity due to driving behavior.

The results indicated that vehicles equipped with one or more DWSs are more likely to be involved in fatal crashes on urban interstates and freeways. Vehicles equipped with DWSs are safer during wet or snowy road conditions, when a vehicle skids laterally or longitudinally, and at intersections than vehicles without DWSs. In addition, vehicles with one or both DWSs are less likely to be involved in drink-and-drive and speeding-related crashes than vehicles without DWSs. For female and elderly drivers, the likelihood of a multivehicle fatal crash occurring while driving a vehicle with one or both DWSs is higher than when driving a vehicle without DWSs, demanding modifications in vehicular technology considering those vehicles.

The modeling results provided valuable insights into the factors influencing fatal crashes involving vehicles with no, one, or two DWSs. The correlated random parameters ordered logit model significantly improves the model accuracy compared to the fixed parameters ordered logit model, indicating that incorporating heterogeneity due to varying driving behaviors is necessary.

The methodological framework proposed in this study provides an overview of various types of unobserved heterogeneity in crash data, along with a step-wise method to incorporate it while modeling. Separate consideration of heterogeneity due to spatial, temporal, and driving behavior variation while modeling provides an overview of the contribution of each aspect of unobserved heterogeneity in model accuracy.

The crash data from 2016 to 2020 were considered for the analysis. The number of crashes involving vehicles with DWSs were increasing, demanding future research at higher penetration of these vehicles to get better insights into factors affecting fatal crashes. Only fatal crash data was used in this research as it is more detailed and includes VIN information. Considering crash data of varying injury severity levels to determine the factors affecting injury severity also merits investigation.

## Disclaimer

This paper is disseminated in the interest of information exchange. The views, opinions, findings, and conclusions reflected in this paper are the responsibility of the authors only and do not represent the official policy or position of the USDOT/OST-R, or any other State, or The University of North Carolina at Charlotte or other entity. The authors are responsible for the facts and the accuracy of the data presented herein. This paper does not constitute a standard, specification, or regulation.

## CRediT authorship contribution statement

**Hardik Gajera:** Data curation, Formal analysis, Investigation, Methodology, Writing – original draft. **Srinivas S. Pulugurtha:** Funding acquisition, Investigation, Methodology, Supervision, Writing – review & editing, Conceptualization.

## Declaration of competing interest

The authors declare that they have no known competing financial interests or personal relationships that could have appeared to influence the work reported in this paper.
